# Healthcare Workers in South Korea Maintain a SARS-CoV-2 Antibody Response Six Months After Receiving a Second Dose of the BNT162b2 mRNA Vaccine

**DOI:** 10.3389/fimmu.2022.827306

**Published:** 2022-01-31

**Authors:** Jae Hong Choi, Young Ree Kim, Sang Taek Heo, Hyunjoo Oh, Misun Kim, Hyang Ran Lee, Jeong Rae Yoo

**Affiliations:** ^1^Department of Pediatric Medicine, Jeju National University College of Medicine, Jeju, South Korea; ^2^Infection Control Unit, Jeju National University Hospital, Jeju, South Korea; ^3^Department of Laboratory Medicine, Jeju National University College of Medicine, Jeju, South Korea; ^4^Department of Internal Medicine, Jeju National University College of Medicine, Jeju, South Korea; ^5^Department of Internal Medicine, Jeju National University Hospital, Jeju, South Korea

**Keywords:** COVID-19, SARS-CoV-2, antibody, neutralizing antibody, Korea

## Abstract

**Background:**

Effective vaccines against coronavirus disease 2019 (COVID-19) are available worldwide; however, the longevity of vaccine effectiveness is not known.

**Objective:**

We performed a prospective observational study to assess the antibody response of healthcare workers against severe acute respiratory syndrome coronavirus 2 (SARS-CoV-2) after BNT162b2 mRNA COVID-19 vaccination.

**Methods:**

SARS-CoV-2 neutralizing antibody (nAb) and spike (S) protein-IgG (S-IgG) antibody titers were examined in participants who received two doses of the BNT162b2 mRNA COVID-19 vaccine in a single center between March 1, 2021, and October 11, 2021. Antibody levels were analyzed at four times: before vaccination (visit 1), 4 weeks after the first vaccination (visit 2), 3 months after the second vaccination (visit 3), and 6 months after the second vaccination (visit 4).

**Results:**

A total of 249 healthcare workers at Jeju National University Hospital were enrolled in this study, and 982 blood samples were analyzed. The mean age was 38.1 ± 9.5 years, and 145 (58.2%) participants were females. Positive nAbs (inhibition rates ≥ 20%) were measured in 166/249 (66.7%) subjects at visit 2, 237/243 (97.5%) subjects at visit 3, and 150/237 (63.3%) subjects at visit 4. A S-IgG (≥50 AU/mL) positivity was detected in 246/249 (98.8%) subjects at visit 1, and all participants had positive S-IgG antibody levels at visits 3 and 4 after being fully vaccinated. Further analysis of S-IgG levels revealed a median quantitative antibody level of 1275.1 AU/mL (interquartile range [IQR] 755.5–2119.0) at visit 2, 2765.9 AU/mL (IQR 1809.8–4138.4) at visit 3, and 970.1 AU/mL (IQR 606.0–1495.9) at visit 4. Patient characteristics, such as age, body mass index, and comorbidity, had no relationship with nAb or S-IgG levels at any of the visits. Considering the change in antibody levels over time, both nAb and S-IgG levels at visit 4 decreased compared with the corresponding levels at visit 3. No evidence of SARS-CoV-2 infection was found among any of the participants throughout the study.

**Conclusions:**

The BNT162b2 mRNA vaccine was effective in protecting healthcare personnel working in COVID-19-related departments. While the level of S-IgG antibodies was maintained for 6 months after the second vaccination, nAb levels waned over this 6-month period, indicating the need for a booster vaccination in some healthcare workers 6 months after full vaccination. Herein, we suggest that further studies are needed to evaluate the need for an interval of booster vaccination after full vaccination.

## Introduction

Coronavirus disease 2019 (COVID-19) vaccinations were initiated on February 26, 2021, in South Korea. The efficacy of vaccines should be evaluated objectively, as it can vary depending on the vaccine type, patient characteristics, and severe acute respiratory syndrome coronavirus 2 (SARS-CoV-2) variants. In a randomized controlled trial, the BNT162b2 mRNA vaccine (tozinameran, Pfizer-BioNTech) showed ≥95% efficacy against symptomatic and severe COVID-19 caused by SARS-CoV-2 (1). In a recent retrospective cohort study in the USA in 2021, the effectiveness of the BNT162b2 mRNA vaccine against SARS-CoV-2 infection was 73%, and that in COVID-19-related hospital admissions it was 90%. In addition, the BNT162b2 mRNA vaccine was highly effective among US healthcare personnel in a real-world setting (2). However, BNT162b2 mRNA vaccine effectiveness against infections caused by the delta variant declined from 93% in the first month following vaccination, to 53% 4 months after full vaccination (3). The vaccine continued to be efficacious in preventing COVID-19 illness and severe disease for more than 5 months (3). In a study in South Korea in March 2021, 289 healthy healthcare workers produced SARS-CoV-2 spike (S) protein antibodies 2 weeks after full BNT162b2 mRNA vaccination (4). In addition, the emergence of the B.1.617.2 (delta) variant of SARS-CoV-2 and the reduced effectiveness over time of the BNT162b2 mRNA vaccine led to a resurgence of COVID-19 cases in individuals that had been vaccinated early (5). Participants who received a booster shot of BNT162b2 mRNA vaccination had 90% lower mortality than participants who did not receive a boost shot (6). In November 2021, the B.1.1.529 (omicron) variant of SARS-CoV-2 was detected as a variant of concern in South Africa, and infections with the omicron and delta variants are rapidly spreading in many countries (7). However, whether the BNT162b2 mRNA vaccine will effectively neutralize infection with the omicron variant remain.

Globally, eight vaccines in phase 3 clinical trials have been approved for emergency COVID-19 use: Pfizer, Moderna, AstraZeneca, Johnson & Johnson/Janssen, Biotechnology, Sinopharm, and Sputnik (8). As of November 18, 2021, 78.6% of the total population in South Korea had been fully vaccinated (9). People need to be inoculated with two dose of the same vaccines as homologous vaccination and homologous prime boost vaccination, respectively, with an interval of 14 days to three months. However, vaccine shortage, efficacy, and adverse event can affect the timing of global herd immunity. Heterologous vaccines may be beneficial for early reduction in the COVID-19 cases, and thus, the current vaccine regimen has been switched to heterologous vaccination with mRNA-based vaccines from homologous vaccination with the **ChAdOx1-S** vaccine worldwide (10). Our hospital, Jeju National University Hospital (JNUH), has been designated as an infectious disease treatment hospital in South Korea, and most healthcare members were vaccinated with the BNT162b2 mRNA vaccine in March, 2021. However, there has been a resurgence of COVID-19 cases because of the delta variant in South Korea (11). Although the preventive efficacy of vaccines has been confirmed in clinical trials, it has not been evaluated at the community level. In addition, it is possible that the efficacy of vaccines will be lower against new strains as they spread globally. Herein, we performed a prospective observational study of healthcare workers at JNUH to assess the SARS-CoV-2 antibody response after the second dose of the mRNA-based BNT162b2 COVID-19 vaccine in a single hospital setting in South Korea.

## Materials and Methods

### Study Design

In this prospective observational study, we analyzed SARS-CoV-2 antibody responses in healthcare workers in COVID-19-related departments prior to and after receiving their first and second dose of the mRNA-based BNT162b2 COVID-19 vaccine in a single hospital designated as an infectious disease treatment center in South Korea. The study was approved by the Institutional Review Board (IRB) of Jeju National University Hospital (IRB file no. 2021-02-010). The patients/participants provided written informed consent to participate in the study.

Participation in the study was open to eligible healthcare workers in the JNUH between March 1, 2021, and October 11, 2021. The participants consisted of doctors, nurses, and technicians from the Departments of Laboratory Medicine and Radiology. JNUH is located on the largest island (33°0 N, 126°0 E) off the coast of the Korean Peninsula. The region has a humid, subtropical climate, and it is warmer than the rest of South Korea (daily mean temperature: 15.8°C–16.6°C). As of August 2021, the total population of Jeju was 697,108, with 675,883 Koreans and 21,225 foreigners (12). There is no land-route connection between the Korean Peninsula and Jeju Island; it is only possible to enter and exit *via* aircraft or water vessel.

**Figure 1:**
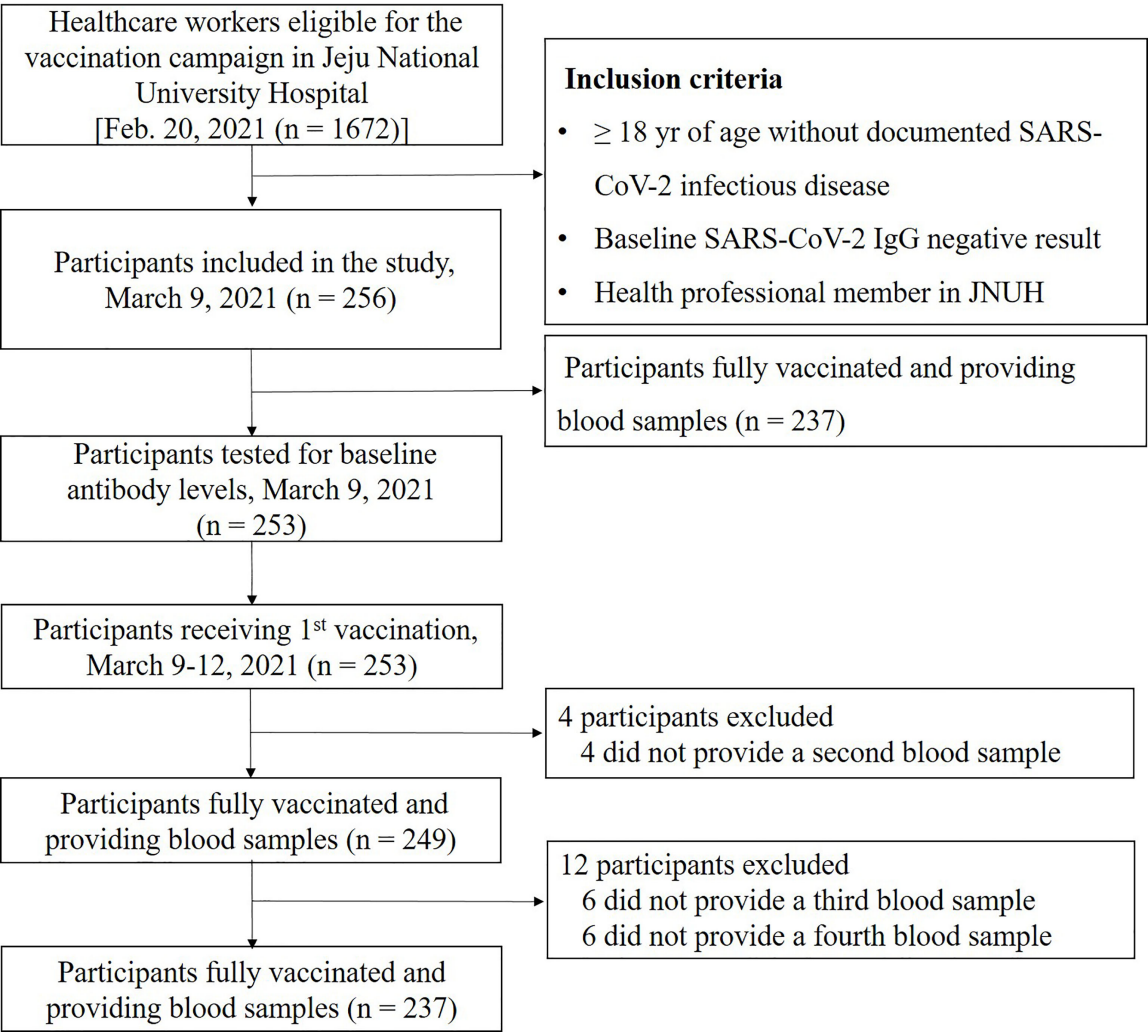
Enrollment and flow of study. This diagram represents all available enrolled participants. Analysis of antibody was performed in 249 subjects who were completed the full vaccination and provided blood samples.

A total of 1672 JNUH healthcare workers were eligible for the SARS-CoV-2 vaccination; of these, 256 subjects who voluntarily agreed to participate in the study were enrolled. Of the 256 participants, 253 provided a blood sample for baseline antibody testing on March 9, 2021, and 249 of these participants showed a negative S-IgG baseline test result and completed the full vaccination schedule (**Figure 1**). Several subjects were excluded because they changed their mind to participate tin the study or were pregnant. Blood samples (10 mL each in three serum-separating tubes) were collected from participants at the JNUH Department of Laboratory Medicine at four times: visit 1 (within 1 week before the first vaccination), visit 2 (4 weeks after the first vaccination), visit 3 (3 months after the second vaccination), and visit 4 (6 months after the second vaccination).

Demographic data were obtained from a standardized questionnaire, which included patient demographics [age, sex, body mass index (BMI)], past medical history and comorbidity index (CCI) score, source of risk exposure to SARS-CoV-2, and the department they worked in. Four characteristics of the participants, age, sex, BMI, and underlying disease, were further analyzed.

### Antibody Detection in Sera

Participant serum samples were sent to the Department of Laboratory Medicine at JNUH to be tested for antibodies against SARS-CoV-2, according to the manufacturer’s instructions (see below). A total of 982 samples (253 from enrolled participants at the baseline visit, 249 from the second visit, 243 from the third visit, and 237 from the final visit) were obtained and analyzed in parallel using the Alinity I SARS-CoV-2 IgG II Quant assay (Abbott Ireland Diagnostics Division, Sligo, Ireland). This is a chemiluminescent microparticle immunoassay used for the qualitative and quantitative determination of IgG antibodies to SARS-CoV-2 in human serum and plasma on the Alinity and ARCHITECT i Systems (9). The SARS-CoV-2 IgG II Quant assay is used to aid in the diagnosis of SARS-CoV-2 infection in conjunction with clinical presentation and other laboratory tests. The assay can also be used to evaluate the immune status of infected individuals and to monitor antibody responses in individuals who have received a COVID-19 vaccine, by quantitatively measuring IgG antibodies against the S receptor-binding domain (RBD) of SARS-CoV-2 (S-IgG). IgG antibody levels < 50 AU/mL were considered nonprotective for this test. The Abbot assay has been validated externally with a sensitivity of 96.77% and a specificity of 99%, with the caveat that these numbers depend on the boundaries of the middle range (10, 13).

In addition, a SARS-CoV-2 neutralizing antibody (nAb) flow immunofluorescent assay (FIA) was used to examine SARS-CoV-2 nAb levels in the Department of Laboratory Medicine at JNUH, according to the manufacturer’s instructions (SD BIOSENSER Suwon-si, Republic of Korea) (10). nAb levels were measured three times (at visits 2, 3, and 4) in this study and analyzed by considering the inhibition rates as semi-quantitative values. Inhibition rates below a PI < 20% were considered nonreactive. The assay was validated with a sensitivity of 100% and a specificity of 99% (14).

### Statistical Analysis

Continuous variables are presented as medians and interquartile ranges (IQR) and compared using the Wilcoxon rank-sum test and Kruskal-Wallis test. Patient comorbidity levels were assessed using the Charlson CCI score for each patient’s previous medical history. Correlation analysis was performed to measure the contributing degree of age and BMI and presented as Pearson’s correlation coefficient. Results were considered statistically significant at a value of *P* < 0.05. Statistical analyses were performed using R software version 3.6.2 (R Foundation for Statistical Computing, Vienna, Austria).

## Results

### Participant Characteristics

During the study period of March 1, 2021, and October 11, 2021, a total of 249 participants were tested after full BNT162b2 mRNA vaccination per protocol (**Figure 1**). The number of participants who completed visits 3 and 4 were 243 and 237, respectively. Baseline characteristics are summarized in **Table 1**. No COVID-19 cases were identified among the total participants during the entire study period; however, one non-participating JNUH healthcare worker was identified with COVID-19 after full vaccination during this study period. The mean age of participants was 38.1 ± 9.5 years, and 145/249 (58.2%) were females. The highest proportion of participants was in the 30–39 year age group (33.7%), followed by the 40–49 year age group (27.7%). The distribution of occupational groups was as follows: health technicians, 91 (36.5%); nurses, 80 (32.1%); and physicians, 78 (31.3%). Among the participants, 43 (17.3%) had underlying diseases, including hypertension (10/43, 23.3%),thyroid disorders (8/43, 18.6%), cerebrovascular disease (4/43, 9.3%), musculoskeletal disorders (4/43, 9.3%), and rheumatologic disorder (2/43, 4.7%).

**Table 1:** Demographic characteristics of the study participants (n = 249).

Variables	Number (%)
Female sex, n (%)	145 (58.2)
Age, yr (mean ± SD)	38.1 ± 9.5
20–29, n (%)	63 (25.3)
30–39, n (%)	84 (33.7)
40–49, n (%)	69 (27.7)
50–59, n (%)	29 (11.6)
60–69, n (%)	4 (1.6)
Occupational group, n (%)	
Physicians	78 (31.3)
Nurses	80 (32.1)
Health technicians	91 (36.5)
Underlying disease, n (%)	43 (17.3)
CCI (mean ± SD)	0.23 ± 0.59

n, number; yr, years; SD, standard deviation; CCI, Charlson comorbidity index score.

### Analysis of nAb Activity by Variable in Fully Vaccinated Participants

**Figure 2:**
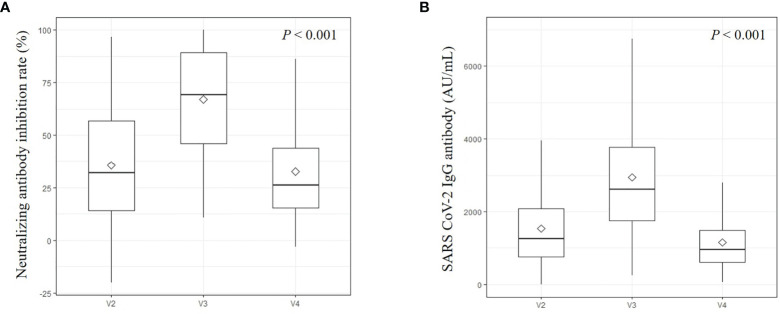
Neutralizing antibody **(A)** and IgG antibodies against the spike receptor-binding domain of SARS-CoV-2 **(B)**. This is an antibody titer analyzed at visit 2 (4 weeks after 1^st^ vaccination), visit 3 (3 months after 2^nd^ vaccination), and visit 4 (6 months after 2^nd^ vaccination). Diamond marks were represented as mean values.

Positive nAb activity (inhibition rates ≥ 20%) was measured in 166/249 (66.7%) subjects at visit 2, 237/243 (97.5%) subjects at visit 3, and 150/237 (63.3%) subjects at visit 4. The median inhibition rate of nAb was 32.2% (IQR 14.2–56.7) at visit 2, 69.2% (IQR 46.0–89.4) at visit 3, and 26.1% (IQR 15.4–43.7) at visit 4 (**Figure 2A**). From visit 2 to visit 3, 29 (11.9%) subjects exhibited decreased level of antibodies, whereas from visit 3 to visit 4, 10 (4.2%) subjects exhibited increased level of antibodies. All these 10 subjects who exhibited increased levels of nAb from visit 3 to visit 4 had decreased S-IgG antibodies during the same period (**Supplementary Figure 1**). Of this group, the mean age was 40.8 ± 10.7 years and the females were 7 (70.0%). The levels of median quantitative S-IgG antibody in this group were 2407.9 AU/mL (IQR 1701.1-3909.5) at visit 3, and 1045.7 AU/mL (IQR 457.4-1257.1) at visit 4. By contrast, only one subject with increased S-IgG had decreased nAb during the same period.

**Figure 3:**
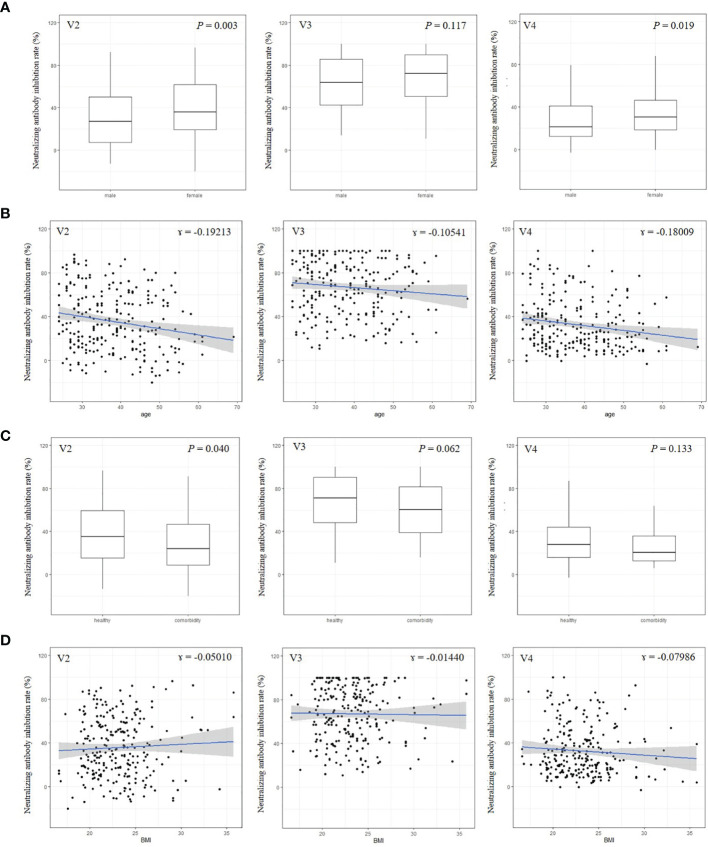
Neutralizing antibody by sex **(A)**, age **(B)**, comorbidity **(C)**, and body mass index **(D)**.This is an antibody titer analyzed at visit 2 (4 weeks after 1^st^ vaccination), visit 3 (3 months after 2^nd^ vaccination), and visit 4 (6 months after 2^nd^ vaccination). BMI, body mass index.

Female subjects tended to have higher nAb titers than males, except at visit 3 (**Figure 3A**). Age and BMI were not associated with nAb titers at any visit (**Figures 3C, D**). Healthy participants had higher levels of nAbs than participants with a comorbidity, but this was statistically significant only at visit 2 (**Figure 3B**).

### IgG Antibodies Against the S-RBD of SARS-CoV-2 in Fully Vaccinated Participants

Among the 249 subjects tested at visit 2, 246 (98.8%) had a meaningful S-IgG antibody level (≥50 AU/mL), and all participants had antibodies at visit 3 and visit 4. The median levels of quantitative S-IgG antibody were 1275.1 AU/mL (IQR 755.5–2119.0) at visit 2, 2765.9 AU/mL (IQR 1809.8–4138.4) at visit 3, and 970.1 AU/mL (IQR 606.0–1495.9) at visit 4 (**Figure 2B**). All measured S-IgG antibody titers at each visit showed significant changes (*P* < 0.001). Twenty-three (9.5%) subjects had decreased S-IgG antibodies from visit 2 to visit 3, and only one (0.4%) subject had increased antibodies from visit 3 to visit 4. Females tended to have higher S-IgG antibody titers than males at each visit (*P* = 0.003, 011, and 0.024, respectively) (**Supplementary Figure 2A**). Relatively young participants (<50 years) had higher S-IgG antibody titers than older participants (≥50 years); however, the tendency was not statistically significant at any visit (ɤ=-0.185, -0.199, -0.179, respectively) (**Supplementary Figure 2C**). Healthy individuals tended to have higher S-IgG antibody titers than participants who had any comorbidity (ɤ=0.016, 012, and 0.036, respectively) (**Supplementary Figure 2B**). Changes in BMI were not associated with changes in S-IgG antibody titers (**Supplementary Figure 2D**).

## Discussion

In this study, we found higher antibody titers, including those of nAbs and S-IgG, after BNT162b2 mRNA vaccination, which was effective in protecting all participants against SARS-CoV-2 during the study period in a real-world setting. In addition, higher nAb and S-IgG antibody titers were observed in participants after the second vaccine dose than after the first vaccine dose. Furthermore, we observed a remarkably higher titer of S-IgG 3 months after the second vaccination than 1 month after the first vaccination. This was followed by a high S-IgG titer that was maintained for 6 months in healthcare personnel working in COVID-19-related departments. However, the mean S-IgG antibody titer decreased 6 months after the second vaccination compared with the titer measured at 3 months. In addition, the proportion of participants positive for SARS-CoV-2 nAbs was 97.5% after 3 months since the second vaccination, declining to 63.3% after 6 months.

The results of this study showed that full vaccination with the BNT162b2 mRNA vaccine maintained nAb and S-IgG levels in participants with more than one risk factor for COVID-19, obese participants, and in adults ≥50 years of age. The SARS-CoV-2 IgG antibody level of vaccinated male participants was lower than that of female participants after full vaccination, which is similar to the results of a study in Israel (15). However, the vaccine effectiveness in other studies was low in adults with more than one risk factor for severe COVID-19 after full vaccination with BNT162b2 mRNA (3, 16, 17). In our study, there was no significant difference in vaccine effectiveness at visit 3 and visit 4 after full vaccination between healthy individuals and those with a comorbidity. Based on these observations, there may be differences in the necessity for booster shots in healthcare workers in regions with a relatively high cumulative prevalence of COVID-19, such as in the USA (14.1% in November 2021) and the United Kingdom (14.2% in November 2021), compared to that in low cumulative prevalence regions, such as observed in South Korea (0.8%) (12).

In a study with full vaccination with the BNT162b2 mRNA in Israel during January and July 2021, the mean period of S-IgG detection after the second vaccination dose was 101 ± 66 days (15); in our study, all participants had S-IgG antibodies 6 months after the second vaccination. As the participants in our study were younger and had a healthier status than the vaccinated population in the Israel study (BMI, 38.1 ± 9.5 vs. 56.5 ± 15.9 and comorbidities 17.0% vs. ≥ 43.0%, respectively), the proportion of participants testing positive for antibodies and the nAb and S-IgG titer levels were higher in our healthcare workers. In addition, the Israeli study reported the highest titer in the first month after the second vaccination [median 9913 AU/mL, IQR (3650–18,733)]; we could not determine this in our study because sequential examination was not performed. In addition, in the Israel study, 16.1% of the subjects had nonprotective S-IgG antibody levels 6 months after BNT162b2 mRNA vaccination, whereas in our study, all participants had a high level of S-IgG antibody levels at 6 months.

Several cohort studies involving healthcare workers have shown the effectiveness of partial vaccination with mRNA vaccines (3, 18). Our study showed that the mean titers of nAbs and S-IgG were relatively high at visit 2 (partial vaccination status) compared with those at visit 4 (six months after full vaccination). However, the differences in partial vaccination in our study should be interpreted with caution owing to the vaccine administration interval being 28 d according to the Korea Disease Control and Prevention Agency recommendation. In addition, the maintenance of a high titer of antibodies in our study was based on relatively young participants with low comorbidities among healthcare workers.

nAbs can directly block viral attachment to target cells by interfering with virus-receptor interactions and by inducing antibody-dependent cellular cytotoxicity, complement-dependent cytotoxicity, and antibody-dependent cellular phagocytosis (19). A previous study showed that COVID-19 vaccinated subjects produced nAbs to SARS-CoV-2 that lasted for 1 year, although with a decrease in titer over time. However, assays performed with serum samples obtained from a small study of participants who had received the BNT162b2 mRNA vaccine showed that the delta variant was 2.9 times more resistant than the B.1 lineage virus in Japan in May 2021 (20). Thus, recent breakthrough SARS-CoV-2 infections in the epidemic era of delta variants in South Korea are a major threat to inhabitants in South Korea, and the delta variant shows pronounced resistance to antibodies elicited by mRNA vaccines and adenovirus vector vaccines. Although a COVID-19 vaccine campaign worldwide helps to slow down the spread of SARS-CoV-2 more effectively and reduce hospitalization and fatalities, antibody levels against SARS-CoV-2 after an initial or second vaccination have been shown to wane in recent studies (3, 21). In addition, the neutralization against the omicron variant was lower than that against the delta variant in participants who had received two and three doses of the BNT162b2 mRNA vaccine (5), and the durability of the effect of the third dose of vaccine against Covid-19 is yet to be determined. However, the results suggest that additional evidence is need *via* further study regarding the interval between second and boost vaccination in healthcare workers after full vaccination. In addition, we suggest that strategies should be developed to determine the timing of a booster shot according to an individual’s condition and based on a test that can measure the nAb or S-IgG antibody titer, especially in response to new SARS-CoV-2 variants.

This study has some limitations. First, this was a single-center study; however, the strength of the study lies in the homogeneity of the cohort because most of the participants were natives of this island. A geographic population exhibits proportionally mixed homogenous and heterogenous characteristics that may cause differences in vaccine effectiveness and population immunity (6). Second, we did not show the peak and then declining slope in antibody levels in vaccinated participants because the levels were not measured continuously. Third, the results of this study are not applicable to patients with a high burden of comorbidity and old age. However, these results provide background data supporting the background for interval of booster shots after full vaccination in high-risk groups in low COVID-19 prevalence countries; healthcare workers are among the highest-risk groups for COVID-19 in South Korea.

In conclusion, the participants in this study received a COVID-19 booster vaccination after the final sample collection (V4) in the fourth week of October 2021, according to the Korea Disease Control and Prevention Agency recommendations at 6 and 8 months after the primary two-dose series with an mRNA-type vaccine made by Pfizer. However, our results revealed that SARS-CoV-2 nAbs and S-IgG were maintained in more than half of the healthcare participants 6 months after being fully vaccinated. Herein, we suggest that further studies are needed to determine whether and/or when a booster shot vaccination is needed, especially for high-risk individuals.

## Data Availability Statement

The original contributions presented in the study are included in the article/**Supplementary Material**. Further inquiries can be directed to the corresponding author.

## Ethics Statement

This study was approved by the institutional review board (IRB) of Jeju National University Hospital (IRB file no. 2021-02-010). The patients/participants provided written informed consent to participate in the study.

## Author Contributions

JY: conceptualization. YK: methodology. HL, HO, and MK: data curation. JY and JC: writing—original draft preparation. SH: supervision. JY and JC: manuscript review. All authors contributed to the manuscript and approved the submitted version.

## Funding

This work was supported by a research grant from Jeju National University Hospital in 2021 [grant number 2021-11].

## Conflict of Interest

The authors declare that the research was conducted in the absence of any commercial or financial relationships that could be construed as a potential conflict of interest.

## Publisher’s Note

All claims expressed in this article are solely those of the authors and do not necessarily represent those of their affiliated organizations, or those of the publisher, the editors and the reviewers. Any product that may be evaluated in this article, or claim that may be made by its manufacturer, is not guaranteed or endorsed by the publisher.

## Supplementary Material

Supplementary Figure 1 Relationship of the change from visit 3 to visit 4 between IgG antibodies against the spike receptor-binding domain of SARS-CoV-2 (shown as S-IgG antibodies) and neutralizing antibody (shown as inhibition rate).

Supplementary Figure 2 IgG antibody against the spike receptor-binding domain of SARS-CoV-2 by sex **(A)**, age **(B)**, comorbidity **(C)**, and body mass index **(D)** in fully vaccinated participants. This is IgG antibody against the spike receptor-binding domain of SARS-CoV-2 analyzed at visit 2 (4 weeks after 1^st^ vaccination), visit 3 (3 months after 2^nd^ vaccination), and visit 4 (6 months after 2^nd^ vaccination).
