# Supplementary figures and images for: Healthcare Workers in South Korea Maintain a SARS-CoV-2 Antibody Response Six Months After Receiving a Second Dose of the BNT162b2 mRNA Vaccine
Source: Front Immunol. 2022 Jan 31;13:827306. doi: 10.3389/fimmu.2022.827306 (PMC8842222; doi:10.3389/fimmu.2022.827306)

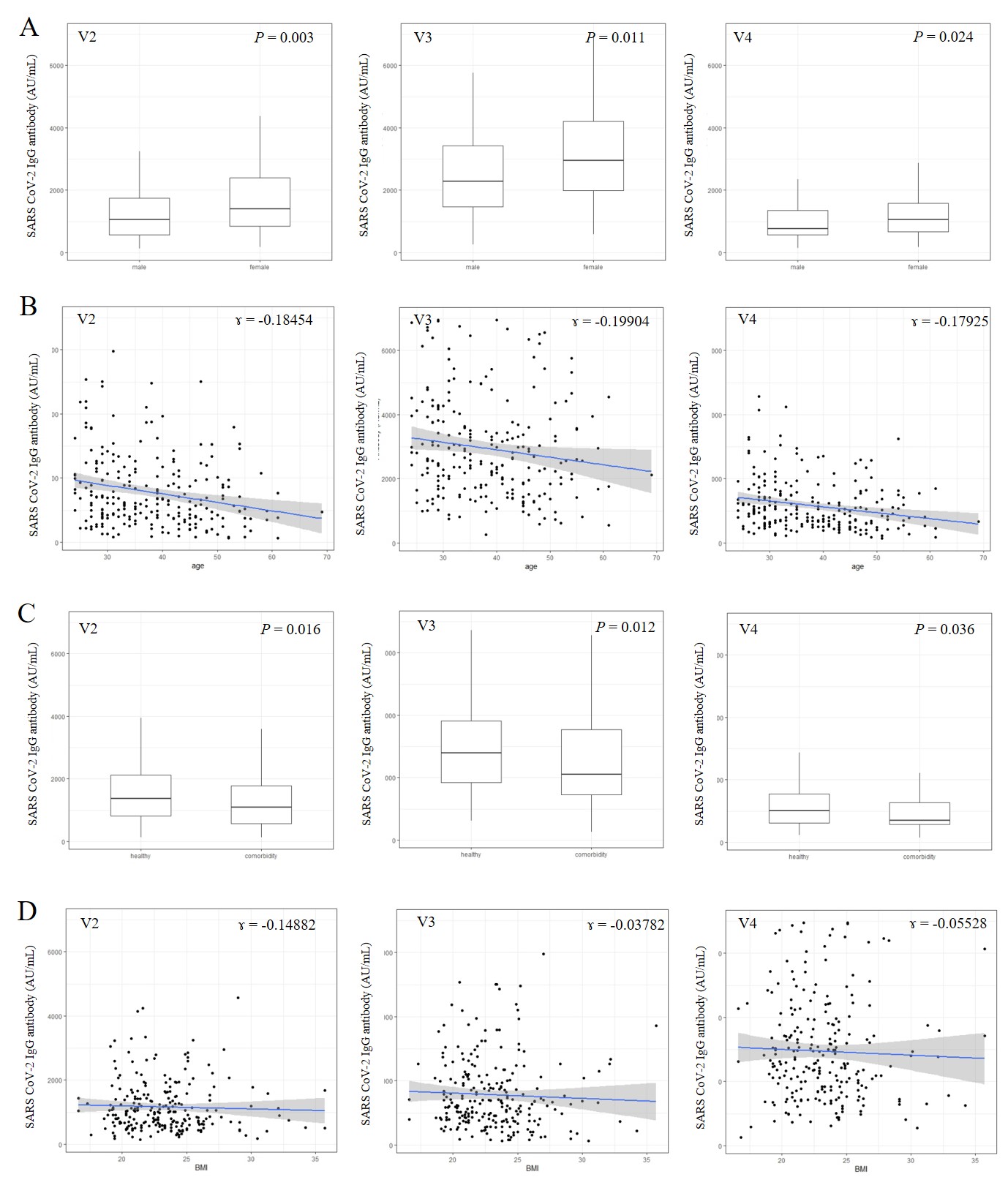

Supplement: Supplementary Figure 1 — Relationship of the change from visit 3 to visit 4 between IgG antibodies against the spike receptor-binding domain of SARS-CoV-2 (shown as S-IgG antibodies) and neutralizing antibody (shown as inhibition rate). [file Image_1.jpg]

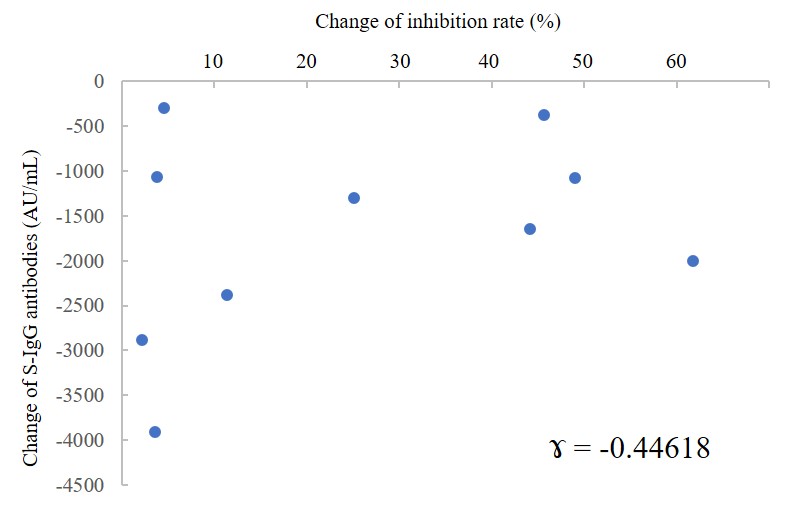

Supplement: Supplementary Figure 2 — IgG antibody against the spike receptor-binding domain of SARS-CoV-2 by sex (A), age (B), comorbidity (C), and body mass index (D) in fully vaccinated participants. This is IgG antibody against the spike receptor-binding domain of SARS-CoV-2 analyzed at visit 2 (4 weeks after 1st vaccination), visit 3 (3 months after 2nd vaccination), and visit 4 (6 months after 2nd vaccination). [file Image_2.jpeg]
